# Patency testing improves capsule retention rates but at what cost? A retrospective look at patency testing

**DOI:** 10.3389/fmed.2023.1046155

**Published:** 2023-08-09

**Authors:** Fintan O'Hara, Caroline Walker, Deirdre McNamara

**Affiliations:** ^1^Trinity Academic Gastroenterology Group (TAGG), Department of Medicine, Trinity College Dublin, Dublin, Ireland; ^2^Department of Gastroenterology, Tallaght University Hospital, Dublin, Ireland

**Keywords:** capsule, endoscopy, patency, small bowel, retention

## Abstract

Capsule retention is one of the major complications of capsule endoscopy, which range from 2.1 to 8.2% depending on the indication. Over the last few years, reported rates of retention have fallen due to better patient selection due to the recognition of risk factors for capsule retention as well as the introduction of the patency capsule. The patency capsule is a dissolvable capsule with the same dimensions as the functional capsule. It breaks down in the GI tract after approximately 30 h, reducing the risk of symptomatic retention. Failure to pass this patency capsule out of the small bowel results in the patient being excluded from capsule endoscopy. We performed a retrospective analysis of the patency capsules performed in our unit over a 12-month period. A total of 166 (14.7%) of 1,127 patients referred for capsule endoscopy were deemed to require patency assessment (45.8% men, mean age 48 years). Of those who passed the patency assessment and underwent capsule endoscopy, no capsule retention was seen. Indication for patency assessment was found to be appropriate in 87.0% (*n* = 147). Overall, the failure rate at the patency assessment was 43.1%. The patency capsule remains an imperfect but useful tool in examining functional patency of the GI tract prior to capsule endoscopy.

## 1. Introduction

Capsule endoscopy (CE) has become an established diagnostic tool in gastroenterology since its introduction in the early 2000s (1). Indications include obscure gastrointestinal (GI) bleeding, assessment and diagnosis of Crohn's disease affecting the small bowel, surveillance in polyposis syndromes, and coeliac disease assessment (2).

Capsule endoscopy benefits from being a well-tolerated non-invasive procedure that is easily performed in an outpatient setting. Adverse events are an infrequent occurrence. The most serious of these potential complications is capsule retention which is defined as “the identification of a capsule on abdominal radiological imaging ≥ 14 days after capsule ingestion, or the need for its surgical removal due to small-bowel obstruction” (3).

A recent meta-analysis showed capsule retention rates of 2.1% for patients with suspected small-bowel bleeding (95% CI 1.5%–2.8%) and 2.2% (95% CI 0.9%–5.0%) for those having evaluation because of abdominal pain and/or diarrhea (4). In suspected IBD, the retention rate was 3.6% (95% CI 1.7–8.6%), while the patient rate with known IBD was 8.2% (95% CI 6.0–11.0%) (4). Although capsule retention is usually asymptomatic and is often passed during follow-up, there is a risk of bowel obstruction and the need for surgery or endoscopic intervention for its removal (5, 6). It remains the feared complication during capsule endoscopy.

The risk factors associated with capsule retention have been well characterized (7). The presence of a combination of obstructive symptoms (abdominal pain, abdominal distension, and nausea/vomiting) before capsule endoscopy, previous small-bowel resections, abdominal/pelvic radiation therapy, and the chronic use of high-dose non-steroidal anti-inflammatory drugs (NSAIDs) have all been shown to increase the risk of capsule retention (8–10).

Rezapour's (4) meta-analysis of capsule retention rates showed retention rates of approximately 2% of patients being investigated for small-bowel bleeding as well as 4% in suspected and 8% in known IBD. This analysis likely overestimates the current retention rate in IBD as it excluded studies that performed patency assessments before CE routinely. The rates also decreased by approximately half in studies that used either a patency capsule (PC) or CT enterography to assess patency before performing CE (4).

Wang's large systematic review of 1,08,079 procedures in 2020 saw a retention rate of 0.73% (7). This rate decreased significantly over the 20-year period examined (coefficient = −0.34%, 95% CI −0.53 to−0.14%, *p* = 0.0006) likely due to the introduction of the PC and improved patient selection as our understanding of risk factors for retention improved. Crohn's disease was the most frequent risk factor associated with retention at 35.41% in this review. Of the 766 retained capsules, surgery was the most frequent intervention (*n* = 352, 45.95%) followed by endoscopic management (*n* = 199, 25.98%). No intervention (*n* = 176, 22.98%) and medical therapy (*n* = 39, 5.09%) made up the remainder (7).

The use of the PC for the evaluation of small-bowel patency in patients with established Crohn's disease is now standard practice and has been endorsed by the European Crohn's and Colitis Organization/European Society of Gastrointestinal Endoscopy guidelines (11).

Capsule endoscopy after failed patency test has a high rate of retention, and 18 patients who had CE after failed patency assessment had a retention rate of 11.1% (12). However, the real capsule was still passed by 89% of those who failed their patency tests.

While it is proper that we err on the side of caution, denying access to capsule endoscopy in a significant proportion of patients unnecessarily, based on a false positive test, could also negatively impact patient care and warrants further examination and the development of strategies to reduce its occurrence. Reflecting this ESGE recommends offering a PC procedure to only those patients with risk factors for retention (3).

Significant progress has been made in reducing rates of capsule retention since the introduction of the procedure over 20 years ago. The present study aimed to retrospectively evaluate the real-world use of the patency capsule to assess its benefits and limitations.

## 2. Materials and methods

In this retrospective study, we evaluated patients who were referred for capsule endoscopy over a period of 12 months from July 2020 to July 2021 in our capsule endoscopy service. All patients referred for capsule endoscopy who underwent patency assessment were included. Patients referred for capsule endoscopy were pre-assessed for risk factors for retention. Risk factors for retention were previous abdominal surgery, known Crohn's disease, long-term or high-dose NSAID use, known GI obstruction, and previous capsule retention. Those with at least one risk factor were sent for patency assessment prior to capsule endoscopy.

The Pillcam Patency Capsule (Medtronic, Dublin, Ireland) was used for the patency assessment. This capsule has the same dimensions as the Pillcam SB3 small-bowel capsule but is instead made of a soluble body consisting of lactose-containing 10% barium sulfate covered with an impermeable film coating and two timer plugs. The intestinal fluid enters *via* the timer plugs, and the capsule begins to dissolve 33 h after oral administration. The retention of the PC in the intestine is designed to result in the complete disintegration of the capsule body leaving behind only the impermeable film coating, which can pass through even narrow stenosis. The Pillcam patency capsule does not contain a radio frequency identification tag, and thus, the confirmation of the capsule remaining in the GI tract requires radiation exposure.

All patients were consented prior to the procedure. The PC was ingested with water. Patients were free to eat and drink as normal before and after PC ingestion. No prokinetic medications or bowel preparations were used as part of the protocol.

The confirmation of functional patency of the GI tract was defined as the PC passed from the body intact within 28 h. Patients who self-reported passage of the intact capsule in their stool within 28 h were deemed to have functional patency. Those who did not report the capsule passage had a plain film abdominal X-ray after 28 h which was examined for the presence of a capsule within the GI tract. If the PC was not observed on the X-ray for 28 h, the intact PC was considered to have been excreted without the patient's knowledge confirming functional patency. Evidence of a capsule remaining in the GI tract on X-ray was deemed as not having functional patency. This conservative approach was due to the difficulty in device localization on a two-dimensional abdominal X-ray. Other imaging techniques, such as abdominal ultrasound, computed tomography (CT), including low-dose CT, and tomosynthesis were not available to assess the PC location in this study.

Patients with confirmed functional patency underwent capsule endoscopy. The primary endpoint was the capsule retention rate in patients who had passed the patency assessment by PC. Secondary endpoints included the rates of confirmed functional patency, by what method patency was confirmed (self reported or *via* radiology), small-bowel transit time on follow-up capsule and adverse events.

The results are reported using descriptive statistics (median and interquartile range) for patient characteristics. Comparisons between groups were made using the Mann–Whitney U-test and Fisher's exact test. Differences at a p-value of <0.05 were considered to be statistically significant.

## 3. Results

### 3.1. Demographics

A total of 1,127 patients who were referred for capsule endoscopy in our unit over a period of 12 months from July 2020 to July 2021 were included.

At pre-assessment, 169 (15%) patients were deemed to have a risk factor for capsule retention and were sent for patency assessment. Of this group, 77 were men (45.6%) with a mean age of 48.2 years (Table 1). This mean was lower than the overall cohort of 1,127 patients who had a mean age of 60 years (SD +/–18.1).

**Table 1:** Demographics.

**Study population demographics**	***n =* 169**
Male, no. (%)	77 (45.6%)
Female, no. (%)	92 (54.4%)
Age (IQR)	48.2 years (34.7 – 64.3)
**Indication for capsule endoscopy**	
Suspected Crohn's disease	84 (49.7%)
Crohn's Assessment	37 (22.5%)
IDA	29 (17.7%)
GI bleeding	4 (2.4%)
Other	13 (7.7%)

The indication for capsule endoscopy in the patency assessment group was a history of Crohn's disease in 49.7% and suspected Crohn's disease in 22.5%. Iron deficiency anemia and GI bleeding were the most common other causes at 17.7 and 2.4%, respectively.

### 3.2. Retention rates

In those patients who passed the patency assessment and underwent capsule endoscopy (*n* = 84), no patient experienced capsule retention. Of these, 82 patients had a complete small-bowel study, while in one patient, the capsule stayed in the stomach for the entire duration of the study and a second passage to the large bowel was not seen. Both had an asymptomatic passage of the capsule on radiological assessment after 14 days.

In our entire cohort of patients who underwent capsule endoscopy during the period of analysis (*n* = 761), only one confirmed case of capsule retention was recorded giving a retention rate of 0.13%. The patient was a 37-year-old man with iron deficiency anemia, no regular medications, and no other medical history of note. This patient did not have a patency test as they were not deemed to have a risk factor for capsule retention on pre-assessment. While asymptomatic from the retained capsule, they underwent surgical resection of a complex small-bowel diverticulum with ulceration which was felt to be the cause of their anemia.

### 3.3. Patency assessment results

Of 169 patients referred for patency, 152 took the procedure. Functional patency was confirmed in 55.3% (*n* = 84) of these. In total, 8.3% (*n* = 7) of patients reported passage of an intact capsule within 28 h, while in 91.7% (*n* = 77), the passage was confirmed *via* abdominal X-ray. There was no statistically significant difference in confirmed patency rates between sexes. Those aged over 60 years had a significantly lower patency rate (41.5% vs. 60.4%, *p* = 0.0442) (Table 2).

**Table 2:** Patency Rates by age and sex.

	**n**	**% of total**	**Confirmed patency**	**Unconfirmed patency**	**Patency rate**	***p*-value**
Male	70	46.10%	42	28	60.00%	
Female	82	53.90%	42	40	51.20%	0.3271
Age </=60	111	73.00%	67	44	60.40%	
Age > 60	41	27.00%	17	24	41.50%	0.0442

Patency rates when analyzed by risk factors for retention at pre-assessment showed some variability; however, the only risk factor to show a statistically significant variation was known as Crohn's disease which interestingly showed a higher patency rate than the remaining indications (72.7% vs. 55.3%, *p* = 0.0292) (Table 3). This we believe is likely due to the selection bias as patients with known complex Crohn's disease are not referred for capsule endoscopy.

**Table 3:** Patency rates by indication.

	**n**	**% of total**	**Confirmed patency**	**Unconfirmed patency**	**Patency rate**	**Univariate analysis, *p*-value**
Previous abdominal surgery	42	27.6%	21	21	50.0%	0.4680
Crohn's disease	33	21.7%	24	9	72.7%	0.0292
NSAIDs	19	12.5%	11	8	57.9%	1.0000
Radiological findings	20	13.2%	7	13	35.0%	0.0573
Endoscopic findings	8	5.3%	4	4	50.0%	1.0000
Obstructive symptoms	9	5.9%	7	2	77.8%	0.1888
Radiation (Abdominal/Pelvic)	2	1.3%	0	2	0.0%	0.205
Suspected Crohn's disease	16	10.5%	9	7	56.3%	1.0000
None documented	3	2.0%	1	2	33.3%	0.5869
Total, n (% of total)	152	100%	84	68	55.3%	

The rates were similar between those with a valid vs. invalid indication for patency assessment using ESGE guidelines (55.6% vs. 52.6%, *p* = 0.8106) (3).

Patients who failed functional patency assessment were excluded from capsule endoscopy and returned to their referring physician for further assessment. Unfortunately, we do not have data on further imaging or endoscopy on these patients.

### 3.4. Indication for patency

When the indication for patency assessment was reviewed, it was found to be appropriate in 87.0% (*n* = 147) when compared with ESGE technical guidelines (3) (Table 4). Of the 13.0% that fell outside guidelines, 81.8% (*n* = 18) of these were for suspected Crohn's disease, while a further 4% had no clear indication documented.

**Table 4:** Indication for patency.

	***n =* 169**	
**Valid Indication**	**147**	**(87.0%)**
Previous abdominal surgery	51	(30.2%)
Known Crohn's disease	37	(21.9%)
Radiological findings	20	(11.8%)
Long term NSAID use	20	(11.8%)
Endoscopic findings	9	(5.3%)
Obstructive symptoms	9	(5.3%)
Previous abdominal radiation	1	(0.6%)
**Invalid indication**	**22**	**(13.0%)**
Suspected Crohn's disease	18	(10.6%)
None documented	4	(2.4%)

### 3.5. Adverse events

Two patients (1.2%) reported abdominal pain and bloating during the patency test. Both were managed conservatively and had passed the PC on radiological assessment at 28 h. Both were excluded from capsule endoscopy and were referred for radiological small-bowel imaging.

### 3.6. Capsule endoscopy results

Overall, 50.0% (*n* = 42) of patients who proceeded to capsule endoscopy following a PC had clinically significant findings on their test (Table 5). Enteritis/ileitis was the most common finding seen in 34.5% (*n* = 29). The mean small-bowel transit time was 241 min (SD +/– 135 min).

**Table 5:** Capsule findings post-patency.

**Normal study**	**42**	**50.0%**
Enteritis/ileitis	29	34.5%
Angiodysplasia	2	2.4%
Duodenitis	3	3.6%
Small-bowel polyp	2	2.4%
Colonic polyp	2	2.4%
Submucosal bulge	1	1.2%
Meckel's diverticulum	1	1.2%
Gastric retention	1	1.2%
Incomplete study	1	1.2%

## 4. Discussion

In this retrospective analysis of patency capsules performed in a single center over a 12-month period, 166 (14.7%) of 1,127 patients referred for capsule endoscopy were deemed to require patency assessment (45.8% men, mean age 48 years).

Of those who passed the patency assessment and underwent capsule endoscopy, no capsule retention was seen. However, the failure rate at the patency assessment was 43.1%.

Short stenoses of the GI tract are difficult to exclude by standard imaging methods. There have been many reported cases of capsule retention in patients who had prior radiological imaging that failed to diagnose short intestinal strictures that were subsequently identified on capsule endoscopy (13). For example, Pennazio et al. reported capsule retention in 5 of 100 patients with obscure GI bleeding who had no small-bowel strictures noted on prior small-bowel follow-through (SBFT) (14). CT scans have also been shown to be poor predictors of capsule retention (15–17). Conversely, no retention of diagnostic capsule endoscopy was seen in 10 patients who had strictures previously confirmed by radiological evaluation (8). As such, the patency capsule has become a recognized gold standard for the assessment of luminal patency prior to capsule endoscopy (18).

In this study, the Pillcam patency capsule was evaluated to determine its real-world utility in reducing capsule retention rates in CE. In our unit with pre-assessment for risk factors for capsule retention, an overall retention rate of 0.14% is low compared to published data. The largest meta-analysis to date showed a retention rate of 2.1 % for patients with suspected small-bowel bleeding (95% CI 1.5–2.8%), 3.6% (95% CI 1.7–8.6%) for suspected IBD, and 8.2 % (95 %CI 6.0–11.0%) in established IBD (4). Appropriate patient selection as evidenced in our study and the use of the PC can significantly reduce retention rates.

All 84 patients in our cohort with confirmation of patency safely underwent capsule endoscopy without retention. Additionally, 50.0% of these patients who underwent the CE procedure had clinically significant findings on their test. Therefore, this study demonstrated that the PC could allow us to carry out CE safely and effectively in patients at an increased risk for capsule retention.

Radiological localization of the PC in those patients who do not self-report passage within 28 h was only available by plain film X-ray. Given the difficulty in accurately localizing within the colon against the small bowel on this modality, any visualized capsule was deemed unconfirmed functional patency. In total, 44.7% of patients failed the patency assessment by these criteria. This compares favorably to a recent meta-analysis with reported failure rates of between 21.4 and 44% were seen (19).

Our data confirm the limitations of the patency test in the setting of limited or no access to three-dimensional imaging to locate the patency capsule accurately. In this setting, strict adherence to ESGE guidelines on the indication for patency assessment will lead to excess numbers of patients being excluded from the test (3).

Preferably, CT or X-ray tomosynthesis would have been used but resource limitations do not allow for their routine usage in our center. Thus, there is likely a high false positive result contained in this 43.1%. Indeed, low-dose CT has been demonstrated to significantly improve localization of the patency capsule compared to plain film X-ray in previous studies[(93.9 vs. 21.2% (*P* < 0.0001)] (20). X-ray tomosynthesis has also demonstrated similar improvements in capsule localization as compared to CT in comparison with abdominal X-ray (21). A recent prospective study using the same patency capsule as this study showed a patency rate of 89.1% where CT was easily accessible (22).

This study has several limitations. The data were retrospectively collected at a single referral center. Guidelines for PC usage were based on ESGE technical guidance; however, there were some patency studies performed which fell outside these criteria or where the indication for PC was unclear. Information regarding further endoscopy or radiology of the patients failing the patency assessment was also not available.

The high false positive rate is likely related to procedural aspects of the test. This warrants further investigation to avoid the unnecessary exclusion of patients from capsule endoscopy as well as indirectly leading to higher costs as patients are directed away from capsule endoscopy to dedicated small-bowel radiological examination and enteroscopy.

Methods to reduce the false positive rate by the use of prokinetics or bowel prep as well as prolonged patency assessment time could be investigated.

It is also important to investigate what happens to patients who fail patency assessments. Depending on the indication for capsule endoscopy, referral to radiology for dedicated small-bowel imaging or proceeding to device-assisted enteroscopy could be considered. Currently, in our tertiary referral center, the decision is done on a case-by-case basis for our own patients where those from external centers are sent back to their primary physician. Protocols should be developed to maximize the diagnostic capability of capsule endoscopy and device-assisted enteroscopy.

## 5. Conclusion

The patency capsule remains the best, albeit an imperfect tool, for examining functional patency of the GI tract prior to capsule endoscopy. The confirmed passage of a PC reduces the risk of capsule retention to an almost negligible level for all indications. However, the high false positive rate remains a limitation. Further prospective research into the procedural aspects of its usage will hopefully reduce this rate in the future.

## Data availability statement

The raw data supporting the conclusions of this article will be made available by the authors, without undue reservation.

## Ethics statement

The studies involving human participants were reviewed and approved by the study was conducted in accordance with the Declaration of Helsinki, and approved by the Ethics Committee of Tallaght University Hospital (protocol number 822, approved on 24 March 2022). Written informed consent for participation was not required for this study in accordance with the national legislation and the institutional requirements.

## Author contributions

FO'H: writing–original draft. FO'H, CW, and DM: writing–review and editing. All authors contributed to the article and approved the submitted version.
